# A time window for rescuing dying retinal ganglion cells

**DOI:** 10.1186/s12964-023-01427-3

**Published:** 2024-01-31

**Authors:** Wenting You, Kèvin Knoops, Iris Boesten, Tos T. J. M. Berendschot, Marc A. M. J. van Zandvoort, Birke J. Benedikter, Carroll A. B. Webers, Chris P. M. Reutelingsperger, Theo G. M. F. Gorgels

**Affiliations:** 1 University Eye Clinic Maastricht UMC+, Maastricht University Medical Center+, Maastricht, 6229 HX The Netherlands; 2 Department of Biochemistry, CARIM School for Cardiovascular Disease, Maastricht University, Maastricht, 6229 ER The Netherlands; 3 Department of Mental Health and Neuroscience, Maastricht University, Maastricht, 6229 ER The Netherlands; 4 The Microscopy CORE lab, Maastricht Multimodal Molecular Imaging Institute, Maastricht University, Maastricht, 6229 ER The Netherlands; 5 Department of Molecular Cell Biology, CARIM School for Cardiovascular Disease, Maastricht University, Maastricht, 6229 ER The Netherlands; 6 Institute of Molecular Cardiovascular Research (IMCAR), Universitätsklinikum Aachen, 52074 Aachen, Germany

**Keywords:** Primary RGCs, Mitochondrial fragmentation, PS exposure, Cytochrome c release, Reversible cell death program

## Abstract

**Background:**

Retinal ganglion cell (RGC) degeneration and death cause vision loss in patients with glaucoma. Regulated cell death, once initiated, is generally considered to be an irreversible process. Recently, we showed that, by timely removing the cell death stimulus, stressed neuronal PC12 cells can recover from phosphatidylserine (PS) exposure, nuclear shrinkage, DNA damage, mitochondrial fragmentation, mitochondrial membrane potential loss, and retraction of neurites, all hallmarks of an activated cell death program. Whether the cell death process can be reversed in neurons of the central nervous system, like RGCs, is still unknown. Here, we studied reversibility of the activated cell death program in primary rat RGCs (prRGCs).

**Methods:**

prRGCs were exposed to ethanol (5%, vol/vol) to induce cell death. At different stages of the cell death process, ethanol was removed by washing and injured prRGCs were further cultured in fresh medium to see whether they recovered. The dynamics of single cells were monitored by high-resolution live-cell spinning disk microscopy. PS exposure, mitochondrial structure, membrane potential, and intracellular Ca^2+^ were revealed by annexin A5-FITC, Mito-tracker, TMRM, and Fluo 8-AM staining, respectively. The distribution of cytochrome c was investigated by immunofluorescence. The ultrastructure of mitochondria was studied by electron microscopy.

**Results:**

Analysis of temporal relationships between mitochondrial changes and PS exposure showed that fragmentation of the mitochondrial network and loss of mitochondrial membrane potential occurred before PS exposure. Mitochondrial changes proceeded caspase-independently, while PS exposure was caspase dependent. Interestingly, prRGCs recovered quickly from these mitochondrial changes but not from PS exposure at the plasma membrane. Correlative light and electron microscopy showed that stress-induced decrease in mitochondrial area, length and cristae number was reversible. Intracellular Ca^2+^ was elevated during this stage of reversible mitochondrial injury, but there was no sign of mitochondrial cytochrome c release.

**Conclusions:**

Our study demonstrates that RGCs with impaired mitochondrial structure and function can fully recover if there is no mitochondrial cytochrome c release yet, and no PS is exposed at the plasma membrane. This finding indicates that there is a time window for rescuing dying or injured RGCs, by simply removing the cell death stimulus.

Video Abstract

**Supplementary Information:**

The online version contains supplementary material available at 10.1186/s12964-023-01427-3.

## Background

Glaucoma is a leading cause of irreversible blindness worldwide with a global prevalence of 3.5% in people aged 40 to 80 years [1]. With the growing number and proportion of the elderly in the population, it is predicted that the number of people with glaucoma worldwide could reach 111.8 million by 2040 [2]. The pathogenesis of glaucoma is multifactorial and highly complex, finally leading to the degeneration and death of retinal ganglion cells (RGCs), the neurons that convey visual information from the retina to the brain [3]. With substantial loss of RGCs, patients experience progressive worsening of vision.

RGCs, like other neurons in the central nervous system (CNS), will not be replaced once they die [4]. The pathogenesis of RGC loss in glaucoma remains incompletely understood. Several cellular processes have been proposed to initiate and accelerate RGC death in glaucoma, including high intraocular pressure [5], neurotrophic factor deprivation [6], mitochondrial dysfunction [7], oxidative stress [8], glutamate excitotoxicity [9], abnormal immune response [10], and vascular dysfunction [11]. Studies indicated that apoptosis may be the final common pathway for RGC death in glaucoma [12]. This cell death program is characterized at the cellular level by cell shrinkage, phosphatidylserine (PS) exposure, mitochondrial fragmentation, chromatin condensation, and caspase activation [13]. Apoptosis is generally considered to be irreversible: once initiated, cells are committed to die. However, recent studies have shown that cells can reverse the process of cell death and recover to normal morphology when the cell death stimulus is removed, even at late stages of apoptosis [14–17]. Reversibility of apoptosis opens up a new, previously unexpected phenomenon in the field of regulated cell death, and may be helpful to rescue cells that are difficult to regenerate, like CNS neurons, such as RGCs.

While for many years, vision loss in glaucoma was considered irreversible, accumulating clinical and preclinical data suggest that vision, at least partially, can recover following lowering of the intraocular pressure (IOP), the major treatment method of glaucoma [5, 18–22]. Experimental studies have shown that acute IOP elevation induces RGC dysfunction measured by electrophysiological tests. This dysfunction was fully reversible by IOP normalization [20, 23]. In a mouse model of chronic ocular hypertension, complete functional recovery was observed after 8 weeks of IOP elevation, but no recovery was evident after 12 weeks of IOP elevation [22]. Clinical studies report visual field improvement in some early glaucoma patients after successful IOP lowering interventions [21, 24]. Taken together, these findings suggest that prior to actual cell death, RGCs may enter a state of physiological dysfunction resulting in impaired visual function. However, these RGCs may recover if the stress that caused the dysfunction is timely relieved. Based on these data, researchers brought up the concept of non-functional, “injured” or “comatose” RGCs [25–27]. However, cellular characteristics of these injured RGCs and the mechanism of recovery are unclear.

The DARC (Detection of Apoptosing Retinal Cells) project has developed a minimally invasive method using fluorescently-labelled annexin A5 to detect rates of RGC apoptosis in situ in glaucoma patients [28]. Annexin A5 detects early apoptotic cells by binding cell surface expressed PS with high affinity [29, 30]. Translocation of PS from the inner to the outer leaflet of the cellular membrane has been shown to be an early event in neuronal apoptosis [31]. Clinical studies also showed that DARC has the potential to identify patients with glaucoma before visual field loss had become evident [32]. Whether annexin A5-labelled, dying RGCs could still recover or be rescued by removing the glaucoma trigger, like high IOP, has not been studied yet.

Recently, we studied the reversibility of the cell death process in in vitro cell death models using various cell death triggers and neuronal cell lines [17, 33]. Ethanol is a widely used cell death trigger [34], which can be easily removed by washing and changing the medium in order to study the reversibility of the cell death process [17]. In the current study, we used ethanol to study the reversibility of the cell death process in prRGCs. At different stages of the cell death process, ethanol was washed away and injured prRGCs were further cultured in fresh medium to see whether they recovered. Special attention was given to the stage of the cell death process in which externalization of PS occurs since this can be elegantly visualized in glaucoma patients by DARC. This study aims to demarcate the phase in the cell death process during which injured or dying RGCs can still be rescued.

## Results

### Survivability of prRGCs after phosphatidylserine exposure

**Fig. 1:**
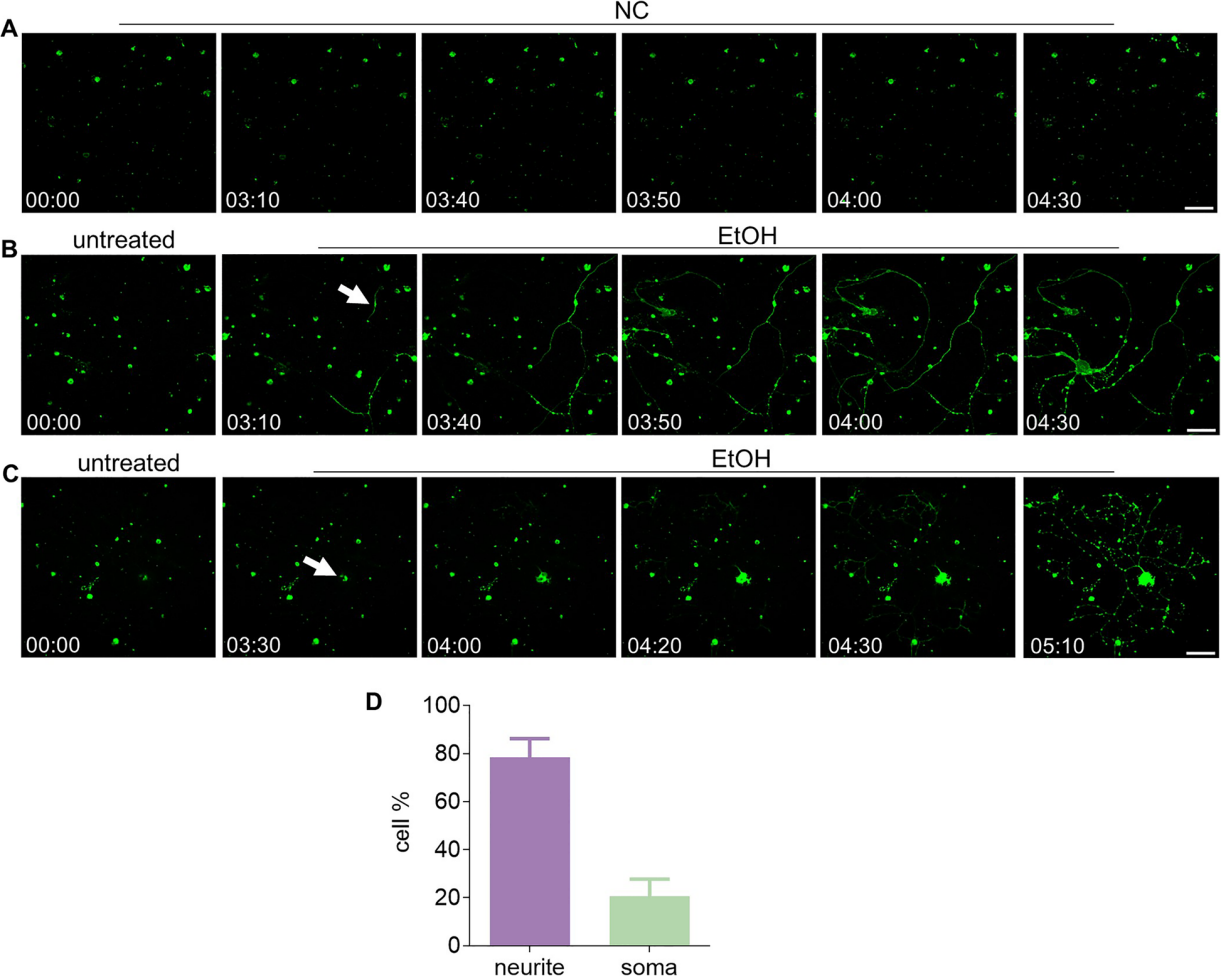
Dynamics of PS exposure in EtOH treated prRGCs. Time-lapse microscopy analysis (6 frames per hour) of the spatial and temporal pattern of PS externalization in rat prRGCs. Cells were imaged with annexin A5-FITC present in the culture medium for the duration of the experiment. Negative control (NC) group showed that staining and imaging were not cytotoxic (**A**). **B **Time-lapse images showing the progressive movement of PS exposure along neurites to soma after EtOH treatment. White arrow points where annexin A5-FITC staining was first observed in neurites. **C **Time-lapse images showing the progressive movement of PS exposure which started from the soma. White arrow indicated the starting point of PS exposure. **D** The percentage of prRGCs in which PS exposure started from neurite or soma. 66 cells were imaged in total. EtOH: ethanol. Scale bar: 50 μm. Time: hr.:min

In the present study, we first explored whether prRGCs could recover from externalization of phosphatidylserine (PS), which has been reported as an early apoptotic marker. In addition, it is an in vivo biomarker for RGC death in glaucoma and a potential biomarker for detection of early stage glaucoma [28, 29]. The spatial and temporal patterns of PS externalization were visualized by annexin A5-FITC staining. Using real-time spinning disk microscopy in living individual prRGCs treated with ethanol (EtOH), we studied annexin A5-FITC binding and observed that either PS exposure originated from a particular location in the neurite and progressed toward the soma (Fig. 1B, Supplemental video 2), or PS exposure originated from the soma (Fig. 1C, Supplemental video 3). Quantitative analysis of these two patterns of PS dynamics showed that in more cases (78 ± 7%) PS exposure originated from neurites than from the soma (22 ± 7%) (Fig. 1D).

**Fig. 2:**
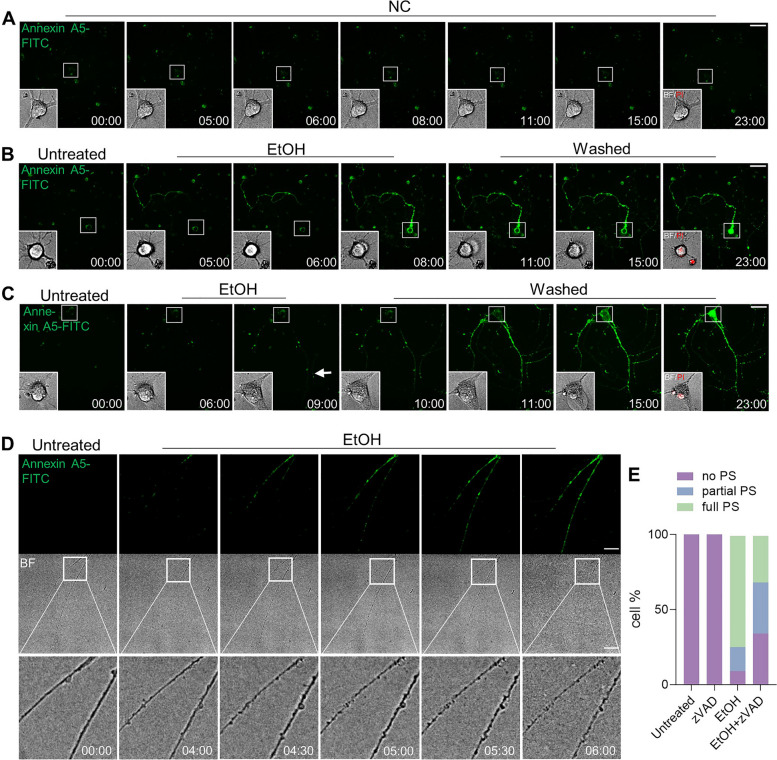
prRGCs cannot survive from PS exposure after removing EtOH. **A** Live cell imaging of individual prRGCs without EtOH treatment (NC, negative control). Cells were imaged with annexin A5-FITC present in the culture medium for the duration of the experiment. Green (annexin A5-FITC) channel of the whole cell and bright field image of cell body were shown. Propidium Iodide (PI, red) was added and imaged at the last time point to detect cell viability. 12 cells were imaged in NC group. Scale bar: 50 μm. **B** Live cell imaging of individual prRGCs before EtOH (5%, vol/vol) treatment (untreated), during EtOH treatment (EtOH), and after washing away EtOH and further culturing cells in fresh culture medium for indicated time (washed). EtOH was removed when annexin A5-FITC staining was observed in the whole cell. Scale bar: 50 μm. **C** Live cell imaging of individual prRGCs before (untreated), during (EtOH), and after (washed) EtOH treatment. EtOH was removed when annexin A5-FITC staining was observed only in neurite. 45 cells were imaged. Scale bar: 50 μm. **D** Live cell imaging to monitor the degeneration of prRGCs neurites along with PS exposure. Scale bar: 50 μm. **E** The percentage of prRGCs with no PS, partial PS, or full PS. prRGCs were pre-treated with Z-VAD-FMK (zVAD, 20 μM) for 1 h, then co-treated with EtOH for another 8 h. 65-80 cells were counted in each group. BF: bright field. EtOH: ethanol. Time: hr.:min

The survivability of prRGCs from externalization of PS was studied by removing EtOH when cells showed annexin A5-FITC staining, and further culturing in fresh culture medium. Figure 2 shows individual prRGCs before (untreated), during treatment (EtOH), and after washing EtOH (washed). The negative control (NC) showed that staining and imaging had no influence on PS exposure, which suggested that imaging and staining was not cytotoxic (Fig. 2A). However, in the EtOH treated cells, no prRGCs survived after removing EtOH as indicated by PI staining of the soma, no matter whether EtOH was removed when the whole cell (Fig. 2B) or only a part of the neurite (Fig. 2C, arrow) was stained with annexin A5-FITC. A previous study reported that axonal degeneration in RGCs was associated with the initiation and propagation of PS externalization [35]. As shown in Fig. 2D, we observed that morphological changes of neurites, like swelling, beading, and fragmentation occurred along with PS exposure. Axon degeneration has been shown to be regulated by the caspase cascades [36, 37]. To verify whether the PS exposure induced by EtOH in prRGCs depended on caspase activation, we used the pan caspase inhibitor Z-VAD-FMK (zVAD) [38, 39] (Supplemental Fig. 1). Comparing the staining patterns of annexin A5-FITC, we classified prRGCs into three different categories: (1) cells with no PS exposure; (2) cells with partial PS exposure (in neurite or soma); (3) cells with full PS exposure. As shown in Fig. 2E, after co-treatment with caspase inhibitor, the percentage of cells with no or partial PS exposure significantly changed. While the percentage of cells without PS exposure increased from 9% in non-pretreated cells to 34% in pre-treated cells (*p* < 0.01), the amount of cell with partial PS exposure increased from 16 to 34% (*p* < 0.05). Cells with full PS exposure decreased from 75 to 32% (*p* < 0.01). Together, these results indicate that the externalization of PS in EtOH-treated prRGCs, from which no cell recovery was observed, probably occurs downstream of caspase activation.

### Temporal relationship between PS exposure and mitochondrial injury

**Fig. 3:**
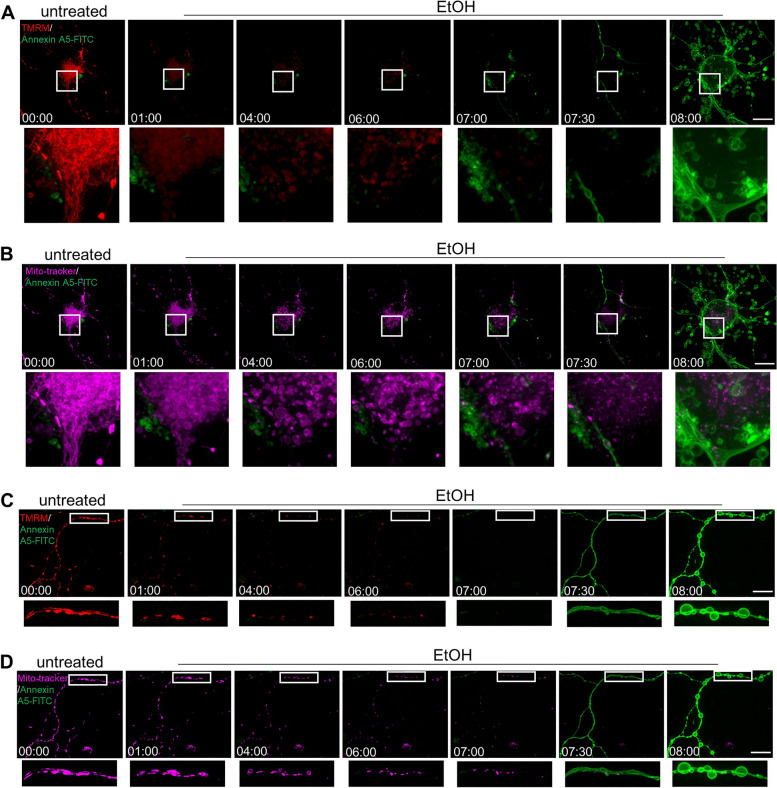
Temporal relationship between PS exposure and mitochondrial injury. Live cell imaging of cell body (**A** and **B**) and neurite (**C** and **D**) of individual prRGCs before (untreated) and during EtOH (5%, vol/vol) treatment (EtOH). PS exposure was detected by annexin A5-FITC (green), mitochondrial membrane potential by TMRM (red), and mitochondrial structure by Mito-tracker (magenta). 29 positions of prRGCs soma and 25 positions with only neurites were imaged. EtOH: ethanol. Scale bar: 20 μm. Time: hr.:min

Mitochondrial fragmentation and membrane potential loss also occur in the process of apoptosis [40]. These mitochondrial changes have been reported to be early events in the process of apoptosis in neuronal cells [41]. We studied the temporal relationship between PS exposure and mitochondrial injury. Figure 3 shows individual prRGCs that were monitored by live cell imaging before (untreated) and after EtOH treatment. Mito-tracker staining was used for imaging the mitochondrial structure, while TMRM staining intensity was used as an indicator of mitochondrial membrane potential. Results showed that in both soma (Fig. 3A, B) and neurite (Fig. 3C, D), mitochondrial fragmentation and membrane potential loss occurred before PS externalization.

### Reversible mitochondrial fragmentation and membrane potential loss

**Fig. 4:**
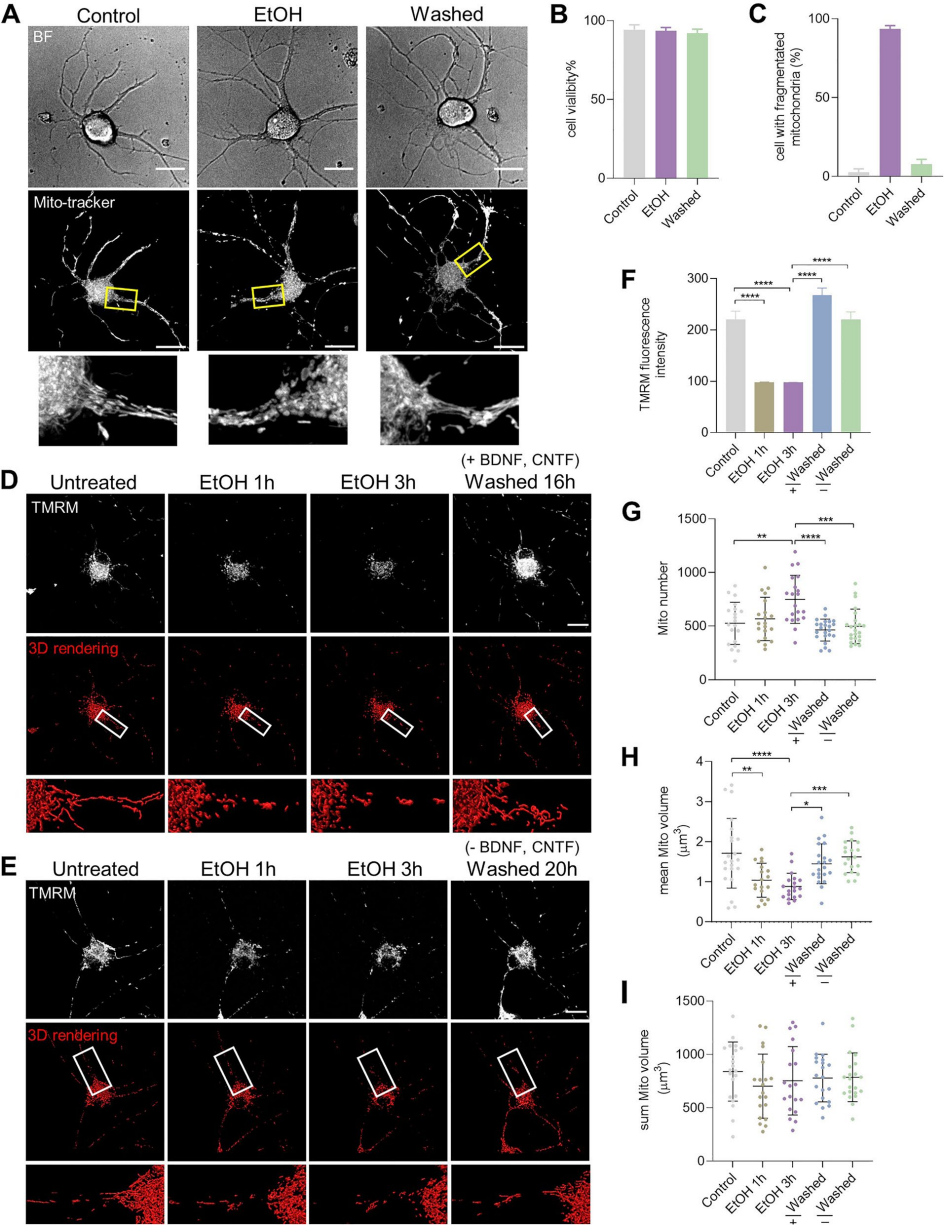
Reversible mitochondrial fragmentation and membrane potential loss in EtOH treated prRGCs. **A** Mito-tracker staining of mitochondria in normal prRGCs, EtOH (5%, vol/vol, 3 h) treated prRGCs, and prRGCs that were EtOH treated and then washed for 20 h (washed). Scale bar: 20 μm. **B** Cell viability was quantified by Hoechst/PI double staining. **C** Percentage of prRGCs with fragmented mitochondria. **D** and **E** The same prRGCs before EtOH treatment (Untreated), treated with 5% EtOH in culture medium for 1 h and 3 h, and then after washing EtOH and further culturing in fresh medium with (+) or without (−) BDNF and CNTF (Washed). Mitochondria were visualized by staining with TMRM. 25 cells were monitored. Scale bar: 20 μm. **F** Quantification of mean fluorescence intensity of TMRM staining. **G**-**I** Quantification analysis of mitochondrial number, mean mitochondrial volume, and total mitochondrial volume in individual prRGCs. Data are presented as the mean ± SD. *p < 0.05, **p < 0.01, ***p < 0.001, ****p < 0.0001. EtOH: ethanol

We then investigated whether prRGCs could recover from this mitochondrial impairment. Mito-tracker staining and representative 3D images from live cell imaging showed that mitochondria normally appeared as a tubular meshwork in normal cells (Fig. 4A). After exposure to EtOH for 3 h, this mitochondrial meshwork fragmented into small, round and swollen mitochondria. Interestingly, after removing EtOH by washing and further culturing cells in fresh culture medium for another 20 h, cells regained a tubular meshwork morphology similar to the morphology observed in non-injured cells. Cell viability showed that there was no significant cell death during EtOH treatment and after removing EtOH (Fig. 4B). Meanwhile, 93% of prRGCs showed mitochondrial fragmentation after EtOH treatment and 92% of these cells regained their normal mitochondrial morphology (Fig. 4C). We further confirmed this reversible mitochondrial fragmentation by monitoring the dynamics of mitochondria in the same individual prRGCs (Fig. 4D). TMRM staining showed that fragmentation of mitochondria was accompanied by loss of membrane potential, which also recovered to normal after removing EtOH (Fig. 4D and F).

### Recovery of mitochondrial fragmentation and membrane potential loss is not dependent on neurotrophic factors

Neurotrophic factors such as brain-derived neurotrophic factor (BDNF), nerve growth factor (NGF), and ciliary neurotrophic factor (CNTF) have been demonstrated to increase neural repair and recovery, promoting neuroprotection and regeneration [42]. Our previous study in neuronal PC12 cells showed that recovery of mitochondrial impairment, including mitochondrial fragmentation and membrane potential loss, did not depend on exogenous NGF [17]. prRGCs are routinely cultured in media that contain the neurotrophic factors BDNF and CNTF. Here, we investigated whether the recovery of mitochondrial impairment in prRGCs depended on these factors by culturing cells in medium without BDNF and CNTF after removing EtOH. We found that cells also recovered their mitochondrial morphology and membrane potential in culture medium without BDNF and CNTF (Fig. 4E and F). By 3D imaging and reconstruction of mitochondrial structure, we further quantified mitochondrial number and volume. Both mitochondrial number and volume varied in individual prRGCs (Fig. 4F-H). The mitochondrial number in individual prRGCs increased from 526 ± 195 to 748 ± 224 (*p* < 0.01) after EtOH treatment for 3 h, recovered to 463 ± 101 (with BDNF, CNTF; *p* < 0.0001) and 496 ± 159 (without BDNF, CNTF; *p* < 0.0001) after washing EtOH (Fig. 4F). The mean volume of individual mitochondria decreased from 1.7 ± 0.8 μm^3^ to 1.0 ± 0.4 μm^3^ (EtOH 1 h, *p* < 0.01) and 0.9 ± 0.3 μm^3^ (EtOH 3 h; *p* < 0.0001), and recovered to 1.5 ± 0.5 μm^3^ (with BDNF, CNTF; *p* < 0.5) and 1.6 ± 0.4 μm^3^ (without BDNF, CNTF; *p* < 0.001) after removing EtOH (Fig. 4G). While these results again showed the mitochondrial fragmentation and its recovery, BDNF and CTNF did not significantly influence this recovery. We found however, no differences in the total volume of all mitochondria in the different groups (Fig. 4H).

### Mitochondrial ultrastructure revealed by CLEM

**Fig. 5:**
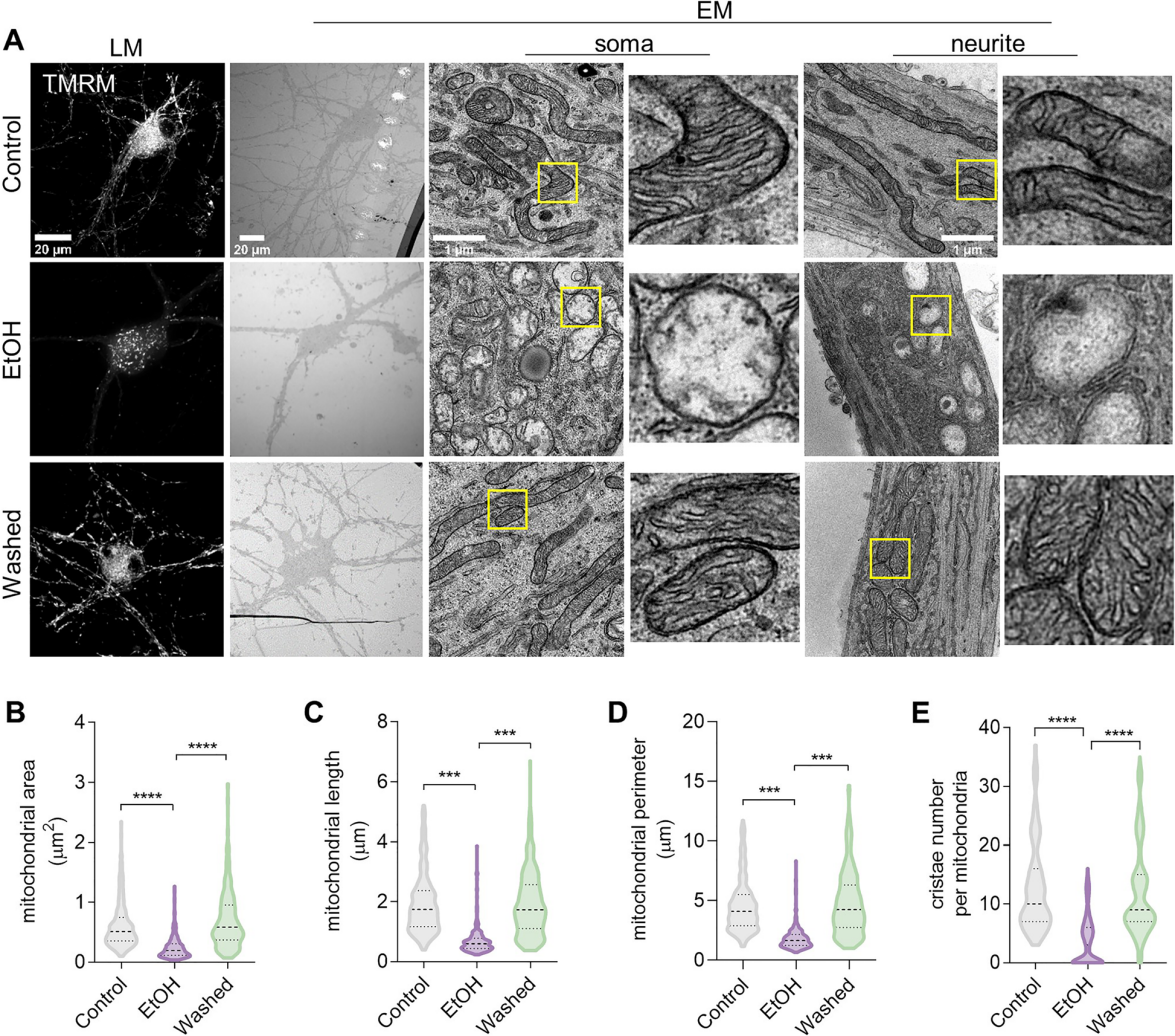
Mitochondrial ultrastructure revealed by CLEM Cells were stained with TMRM for mitochondrial membrane potential and imaged by light microscopy (LM) before preparation for electron microscopy (EM) of the same fields of cells. **A** Representative ultrastructural images of mitochondria from prRGCs’ soma and neurite. **B-E** Quantification of mitochondria related parameters, including mitochondrial length, perimeter, area, and cristae number per mitochondrion. At least 300 mitochondria were quantified in each group. Data are presented as the mean ± SEM. ***p < 0.001, ****p < 0.0001. EtOH: ethanol

We next looked into the ultrastructure of mitochondria in prRGCs, since this varies considerably in relation to the cell’s physiological and pathological state [43]. As shown in Fig. 5A, the mitochondria in the control group with normal membrane potential mainly appeared with a tubular or round profile in cross sections of both soma and neurite. The inner membrane is enfolded perpendicular to the longitudinal axis to form a moderate number of cristae. In prRGCs treated with EtOH for 3 h, which showed reduced mitochondrial membrane potential, mitochondrial structure was significantly damaged in comparison with control group. Mostly, these damaged mitochondria had a small round or oval structure with a low matrix density, exhibited reduced or damaged cristae that were broken, shorter, or highly swollen. Notably, in the washed group of prRGCs, which regained normal mitochondrial membrane potential, the normal mitochondrial structure was apparent, showing the elongated tubular morphology with zigzag cristae and dense matrix. Quantitative analysis showed significant decrease of mitochondrial area (0.24 ± 0.01 μm^2^, *p* < 0.0001; Fig. 5B), length (0.69 ± 0.03 μm, *p* < 0.001; Fig. 5C), perimeter (1.87 ± 0.06 μm, *p* < 0.001; Fig. 5D), and cristae number per mitochondrion (3.59 ± 0.24, *p* < 0.0001; Fig. 5E) in EtOH treated prRGCs compared with control group (0.61 ± 0.02 μm^2^ of area; 1.94 ± 0.06 μm of length; 4.59 ± 0.13 μm of perimeter; 12.86 ± 0.44 of cristae number). In the washed group, all four mitochondrial parameters mitochondrial area (0.72 ± 0.03 μm^2^, *p* < 0.0001), length (1.94 ± 0.06 μm, *p* < 0.001), perimeter (4.79 ± 0.15 μm, *p* < 0.001), and cristae number (11.87 ± 0.43, *p* < 0.0001) recovered to levels similar to those in untreated cells (Fig. 5B-E).

### Reversible mitochondrial fragmentation was accompanied by elevation of intracellular Ca^2+^ but occurred before cytochrome c release

**Fig. 6:**
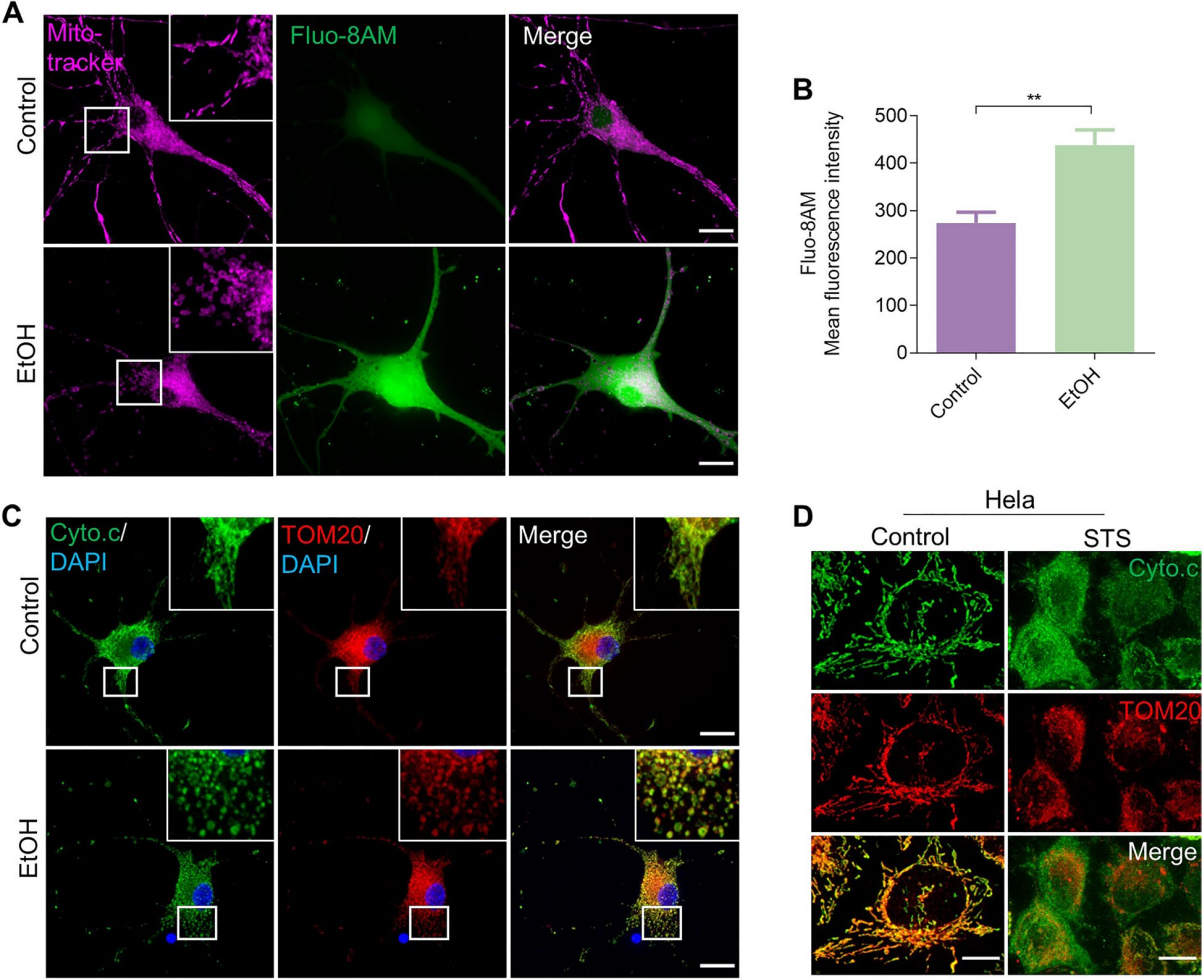
Reversible mitochondrial fragmentation was accompanied by elevation of intracellular Ca^2+^ and occurred before cytochrome c release. **A** Live cell imaging of untreated and EtOH (5%, vol/vol, 3 h) treated prRGCs. Cells were stained with Mito-tracker (magenta) to show mitochondrial structure and Fluo-8 AM (green) to indicate intracellular Ca^2+^ level. Scale bar: 20 μm. **B** Quantification of intracellular Ca^2+^ mean fluorescence intensity in EtOH treated prRGCs. Data are presented as the mean ± SEM. **p < 0.01. **C** Immunofluorescence images of fixed prRGCs after EtOH treatment for 3 h. Mitochondria were stained by TOM20 antibody (magenta), cytochrome c by Cyto.c antibody (green), and nuclei by DAPI (blue). Scale bar: 20 μm. **D** Immunofluorescence images of fixed Hela cell after STS (500 nM) treatment for 2 h. Mitochondria were stained by TOM20 antibody (magenta) and cytochrome c by Cyto.c antibody (green). Scale bar: 10 μm

Mitochondrial morphology is closely associated with the ability of mitochondria to maintain redox balance and Ca^2+^ homeostasis, and mediate the mechanisms of cell death [44]. We further investigated which other cellular injuries had happened before prRGCs could recover from mitochondrial fragmentation and membrane potential loss. By staining cells with Fluo-8 AM, a fluorescent dye of intracellular Ca^2+^, we found that there was a significant increase of intracellular Ca^2+^ concentration after EtOH treatment for 3 h (*p* < 0.01), during the reversible mitochondrial fragmentation (Fig. 6A and B).

Mitochondria mediate apoptosis by mitochondrial outer membrane permeabilization (MOMP), which has been considered as the ‘point of no return’ in the cell death process. Following MOMP, cytochrome c is released from mitochondria into the cytosol where it triggers activation of effector caspases and ultimately leads to apoptotic cell dismantling [45]. In order to study whether cytochrome c had been released from mitochondria at the stage of reversible mitochondrial impairment, we visualized the distribution of cytochrome c by immunostaining cells with anti-cytochrome c antibody. The morphology of mitochondria was revealed by immunostaining cells with anti-TOM20 antibody. A positive control of cytochrome c release was performed first in staurosporine (STS) treated HeLa cells, an apoptotic cell death model [46]. As shown in Fig. 6D, in normal HeLa cells, cytochrome c was mainly distributed in mitochondria, which had the shape of the tubular meshwork. After exposing to STS for 3 h, TOM20 immunostaining showed fragmented mitochondria, while the cytochrome c signal was diffused significantly into the cytosol. In contrast, after treating prRGCs with EtOH for 3 h, when cells showed significant mitochondrial fragmentation indicated by TOM20, cytochrome c showed a similar punctuated immunostaining. No indications of diffusion of cytochrome c into the cytosol were observed.

**Fig. 7:**
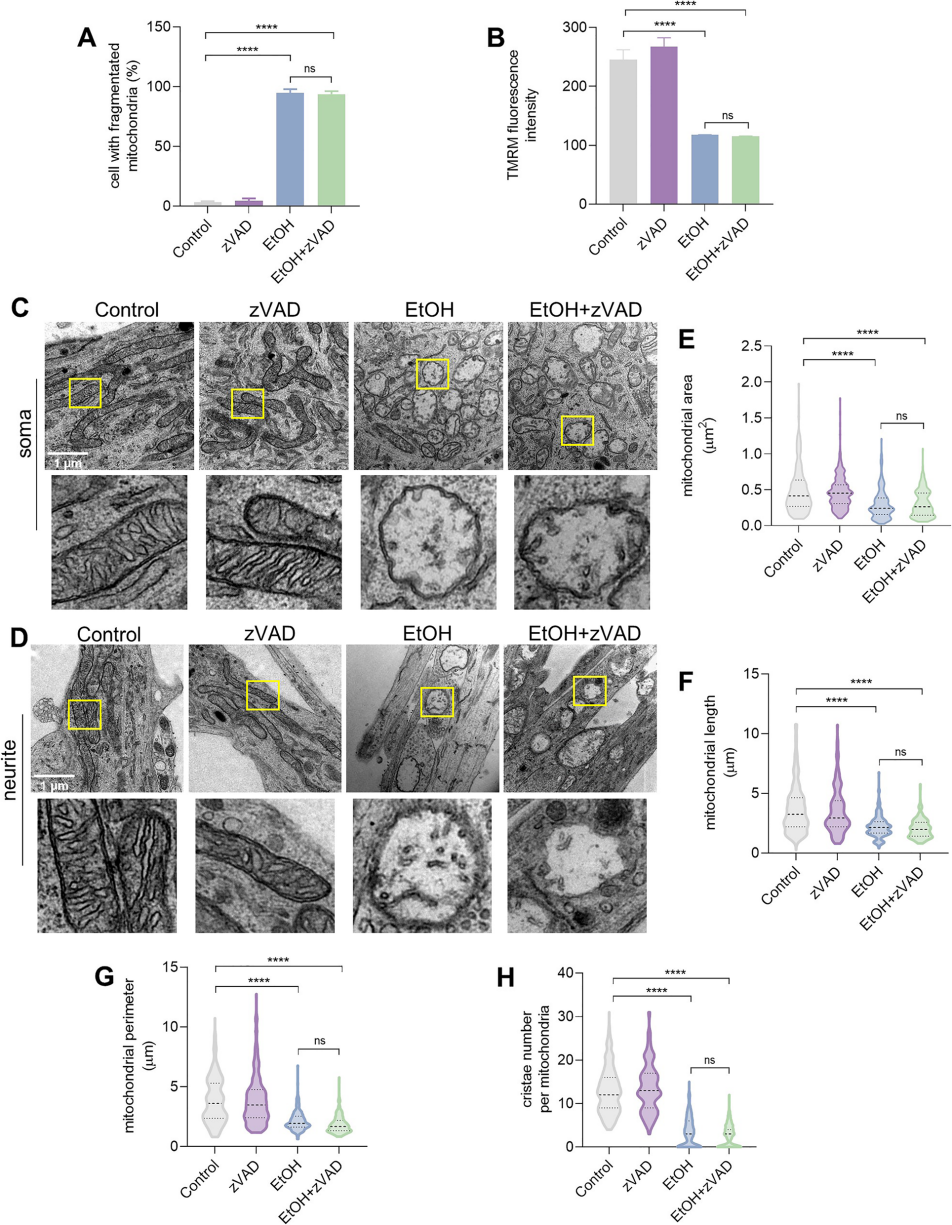
Mitochondrial impairment cannot be inhibited by caspase inhibitor. prRGCs were treated with zVAD (20 μm, 4 h) or EtOH (5%, vol/vol, 3 h), or pretreated with zVAD for 1 h and then co-treated with EtOH for another 3 h. **A** Percentage of cells with fragmented mitochondria. **B** Quantified mitochondrial membrane potential by the fluorescence intensity of TMRM staining in living prRGCs. **C** and **D** Representative ultrastructure images of RGC mitochondria in soma and neurite. Scale bar: 1 μm. **E**-**H** Quantification of mitochondrial area, length, perimeter, and cristae number per mitochondria. At least 300 mitochondria are quantified in each group. Data are presented as the mean ± SEM. ****p < 0.0001. EtOH: ethanol

We also examined the possible involvement of caspase activation at the stage of reversible mitochondrial fragmentation by conducting the experiment in the presence of general caspase inhibitor zVAD. The results showed that zVAD treatment neither inhibited EtOH induced mitochondrial fragmentation (*p* > 0.05) (Fig. 7A) nor membrane potential loss (*p* > 0.05) (Fig. 7B). Electron microscopy showed that zVAD also did not inhibit EtOH induced mitochondrial ultrastructure damage in both soma (Fig. 7C) and neurite (Fig. 7D) of prRGCs, such as fragmentation, swelling, damaged cristae, and decreased matrix density. Quantitative analysis showed no significant difference in mitochondrial area, length, perimeter or cristae number between the EtOH group and co-treated group with zVAD (Fig. 7E-H).

### Mitochondrial ultrastructure of prRGCs with PS exposure

**Fig. 8:**
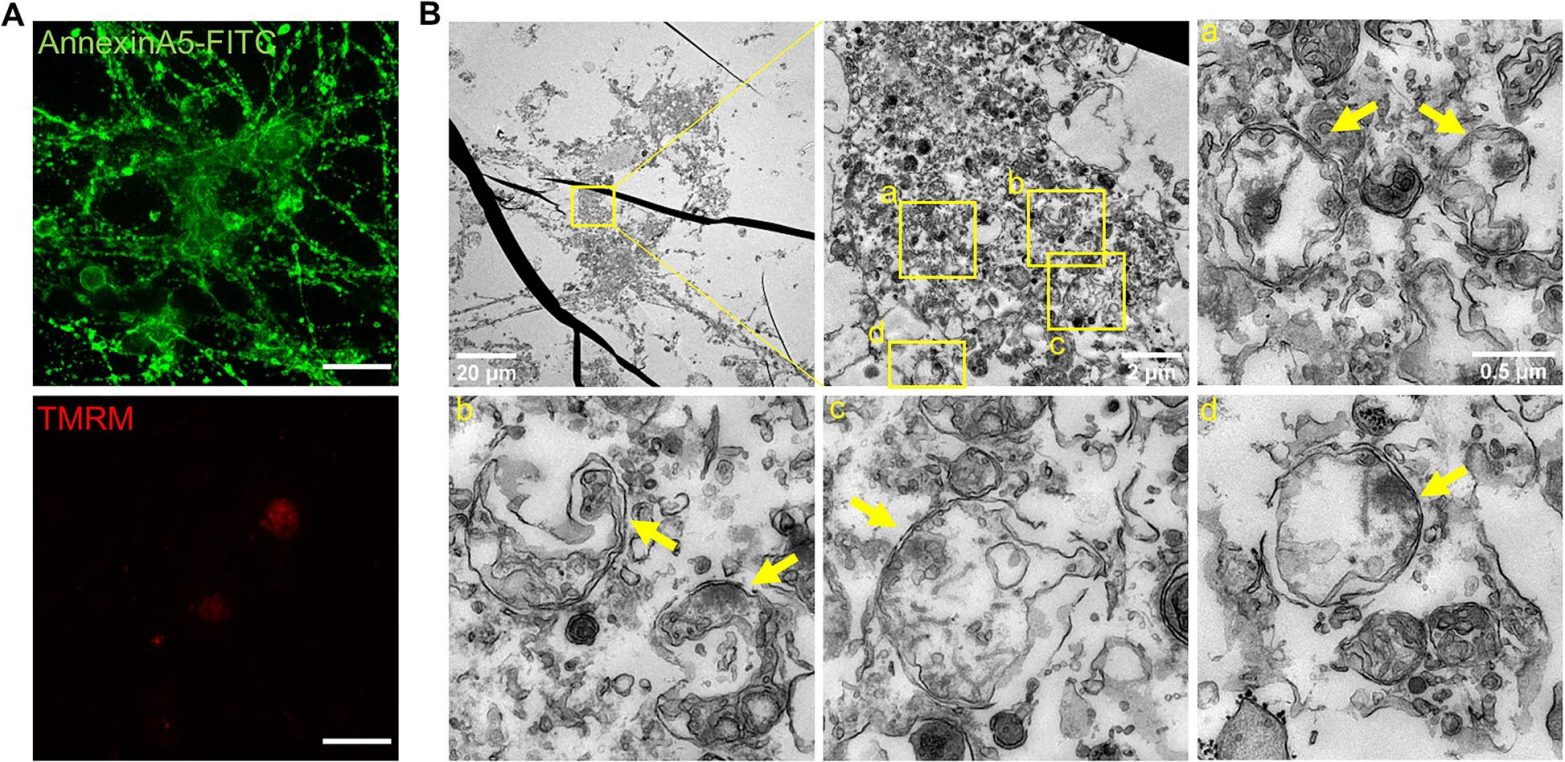
Mitochondrial ultrastructure of prRGCs with PS exposure. **A** Cells were treated with EtOH (5%, vol/vol, 7 h) and imaged by fluorescence microscopy. PS exposure was detected by annexin A5-FITC (green) staining, mitochondrial membrane potential by TMRM (red). Scale bar: 20 μm. Then cells were fixed and re-localized by electron microscopy (**B**). Electron micrographs of the boxed regions are shown in panels a - d. Yellow arrows point at mitochondria

Next, we asked what the ultrastructure and damage of mitochondria is in cells that show PS exposure and cannot survive after removing the cell death stimulus. By using CLEM, in EtOH treated prRGCs that showed reduced mitochondrial membrane potential and were fully stained with annexin A5-FITC, we observed an extensively damaged ultrastructure of mitochondria, which showed not only fragmentation, disappeared cristae, lower matrix density, but also broken inner and outer mitochondrial membranes (Fig. 8).

## Discussion

RGCs, like other neurons in the central nervous system (CNS), have limited regeneration capacity and are generally not replaced once they die. In the present study utilizing purified primary rat RGCs, our results indicate there is a time window in the cell death process during which injured or dying RGCs can recover. Recovery was possible during the stage when RGCs showed mitochondrial fragmentation and mitochondrial membrane potential loss accompanied with elevation of intracellular Ca^2+^. This stage occurred before the stage of massive mitochondrial cytochrome c release. When caspase dependent PS externalization occurred, RGCs could no longer recover.

The death of RGCs is the main cause of the irreversible vision loss in glaucoma [47]. Both clinical and experimental evidence indicate that apoptosis may be the final common pathway for RGC death [48–50]. The apoptotic process requires the expression of specific genes and is characterized by specific cell death events [13]. Recent studies reported that in various cell types, removal of cell death stimulus could stop the cell death process and lead to recovery from diverse apoptotic events that occur at different stages of apoptosis. These, reportedly reversible stages include PS exposure to the outer leaflet of the plasma membrane, mitochondrial fragmentation, nuclear condensation, cytochrome c release, and caspase activation [15, 16, 34]. PS externalization has been reported to be an early marker of neuronal apoptosis, which can be detected by taking advantage of the PS-binding property of fluorescently labelled annexin A5 [31]. In glaucoma, the DARC (Detection of Apoptosing Retinal Cells) project has developed a minimally invasive method using fluorescently-labelled annexin A5 to detect rates of apoptosis in RGCs [28]. In the current study, we investigated reversibility of the cell death program in an in vitro RGCs death model with a focus on the stage of PS translocation. We first observed two kinds of PS exposure dynamics: originating from a location on the neurite of RGCs and progressing toward the soma, or originating from the soma (Fig. 1). The dynamics of PS exposure in the process of RGCs degeneration have been previously investigated, with findings indicating that when the neurite was injured locally, PS exposure starts at the injured neurite and progresses toward the soma [35]. In our study using EtOH as a stimulus, which is applied to the whole cell, we extended these observations by identifying PS exposure originating from the soma as well. This suggests that different triggers and different sites of application may lead to diverse patterns of PS exposure. From both kinds of PS exposure, no RGCs survived in our study after removing the cell death stimulus (Fig. 2A-C). It has been shown that there are two distinct pathways regulating PS exposure: one is a calcium-dependent, caspase-independent pathway, which is reversible; the other is caspase-dependent, which leads to irreversible PS exposure [37, 51–53]. Our results with the caspase inhibitor zVAD showed significant inhibition in EtOH induced PS translocation in RGCs, indicating that PS exposure in this cell death model may be downstream of caspase activation and at least partly dependent on caspase. We also observed neurite degeneration along with PS exposure (Fig. 2D), consistent with a previous study [54]. Taken together, our findings suggest that in our acute cell death model, RGCs recovery is not possible when cells have entered the stage of apoptosis that involves caspase activation. To predict whether PS exposed RGCs that are detected by DARC in vivo can be rescued or not, further studies in experimental glaucoma animal model are needed, investigating the mechanism of PS exposure in situ, also in a chronic as opposed to acute model.

Mitochondria have been recognized as highly dynamic structures undergoing changes in shape and volume resulting in a mixed population of interconnected tubular meshwork and small individual spherical mitochondria within cells [55]. Mitochondria are not only the main site of cellular energy production, but also play critical roles in cell cycle progression, immune responses, calcium homeostasis, and apoptotic cell death [56]. Disturbed mitochondrial dynamics are connected to a variety of diseases that are characterized by impaired mitochondrial function and increased cell death [57]. Some reports indicated that mitochondria undergo rapid, extensive fragmentation early in the apoptotic process, resulting in smaller, rounder mitochondria [58, 59]. In our study, by comparing the temporal relationship between PS exposure and mitochondrial injury in EtOH treated RGCs, we found that mitochondrial fragmentation and membrane potential loss occurred earlier than PS exposure (Fig. 3). Moreover, we observed that mitochondrial fragmentation and membrane potential loss could spontaneously recover to levels similar to those of non-injured RGCs (Figs. 4 and 5, Supplemental video 3). Recent evidence from our group demonstrates that reversible mitochondrial fragmentation is possible in various neuronal cell lines [33], as well as in multiple other cell types treated with diverse cell death stimuli as has been reported by others before [34, 60]. Collectively, these findings suggest that recovery from mitochondrial fragmentation and membrane potential loss is a general phenomenon in the cell death process.

Histologic and ultrastructural findings in patients with glaucoma and experimental glaucoma models showed mitochondrial damage including fragmentation, swelling, disruption of the cristae, and dilution of the matrix [61, 62]. However, whether these deranged mitochondria could still recover to a normal mitochondrial network has not been reported. In our current study, we observed similar changes in mitochondrial ultrastructure in rat RGCs as reported. Importantly, these damaged mitochondria could regain their tubular network with normal cristae and matrix density (Fig. 5). The shape of mitochondrial cristae determines the stability of mitochondrial respiratory efficiency [63]. Interruption of intracellular Ca^2+^ homeostasis has been reported to induce dramatic alterations in cristae topology and metabolic defects [64]. Our results also showed that reversible mitochondrial fragmentation was accompanied by elevation of intracellular Ca^2+^ (Fig. 6). Furthermore, during apoptosis, remodeling of the curvature of the cristae membrane is required for the complete release of cytochrome c, which is normally confined to the cristae [65]. Cytochrome c release follows mitochondrial outer membrane permeabilization (MOMP), which is usually considered to be the ‘point of no return’ in the cell death process [45]. Of interest, the reversible mitochondrial changes (fragmentation and membrane potential loss) occurred before mitochondrial cytochrome c release (Fig. 6). At the irreversible stage of the cell death program when RGCs showed caspase dependent PS exposure, the ultrastructure of mitochondria showed ruptures of the inner and outer mitochondrial membrane (Fig. 8). A previous study also revealed that mitochondria which had released cytochrome c and lost membrane potential showed vesicular and swollen structures with broken outer membrane and this extent of mitochondrial damage appeared to reflect active caspase [66]. In our study, by using the general caspase inhibitor zVAD, we found that zVAD did not inhibit EtOH induced mitochondrial fragmentation, membrane potential loss, and corresponding ultrastructural changes (Fig. 7), which indicated that caspase had not been activated or was not involved at this stage of reversible mitochondrial injury. Above all, our findings indicated that dying or injured RGCs can be rescued even if cells had clear but limited mitochondrial damage. However, RGCs did not recover from the stage of massive cytochrome c release, caspase dependent PS externalization and the corresponding more severe mitochondrial ultrastructural damage.

Neurotrophins are diffusible trophic molecules that exert a potent survival effect on adult CNS neurons undergoing degeneration. These include NGF, BDNF, CNTF, neurotrophin-3 (NT-3) and neurotrophin-4/5 (NT-4/5) in mammals [42]. RGCs can receive neurotrophins that are locally produced in the retina, or that are retrogradely transported from the brain [67]. BDNF and CNTF are present in the standard culture medium of primary rat RGCs, to promote the differentiation, axon growth, as well as survival of RGCs after isolation. We found that the recovery of mitochondrial structure and function was not dependent on providing these neurotrophins in the medium (Fig. 4). However, we cannot rule out the possibility that the primary rat RGCs themselves produce and secrete BDNF or other neurotrophins which may have contributed to the recovery.

Mitochondrial biogenesis and structure are highly influenced by the needs of the cell. In particular RGCs with high energy consumption have an abundance of mitochondria in their soma and axons to produce the necessary ATP to propagate action potentials to the brain [68]. There is substantial evidence that mitochondrial dynamics, as well as ultrastructure and volume, are mechanistically linked to the pathogenesis of glaucomatous degeneration [69]. Details of the intricate mechanisms of mitochondrial dynamics, like fission, fusion and biogenesis, have been explored comprehensively. Important regulators in mammalian cells include mitofusins1 and 2 (Mfn1, 2), optic atrophy-1 (OPA1), dynamin-related protein 1 (Drp1), and peroxisome proliferator-activated receptor gamma-coactivator 1-alpha (PGC-1α) [70]. Fragmented and injured mitochondria can be degraded by mitochondrial specific autophagy, i.e. mitophagy. Increased mitophagy in the retina has been observed in a mouse glaucoma model that is ultimately neuroprotective [71]. Theoretically, all these molecular and cellular activities may be involved in the process of recovery of mitochondrial structure and function in RGCs. However, the exact molecular mechanism has not been identified and further studies are needed.

Research on RGCs in glaucoma has been focused on three aspects: neuroprotection, neuroregeneration, and neurorescue. Neurorescue targets at RGCs that are damaged, dying but not dead, and can be revived [26]. It used to be a general clinical dictum that visual loss from glaucoma cannot be reversed. Accumulating clinical and preclinical data point to reversible RGCs dysfunction and demonstrate that vision recovery in glaucoma patients can occur after IOP reduction [25, 72]. These findings suggest that prior to cell death, RGCs enter a state of physiological dysfunction resulting in impaired visual function, from which cells can recover if the cell death stimulus is taken away timely. Our in vitro model, using purified prRGCs, provides evidence that RGCs undergoing cell death can survive and recover upon removal of the cell death stimulus. This insight may contribute to a better understanding of reversible RGC dysfunction and the observed vision recovery in both animal glaucoma models and clinical settings. However, it is important to notice limitations in our study. First, glaucoma manifests in both acute and chronic forms, with the majority of cases characterized by a slow, progressive degenerative process. Our model is an acute cell death model. It is uncertain whether this accurately replicates the gradual degenerative processes that may occur in vivo. To establish this, comparison of our findings with those of chronic glaucoma animal models would be valuable, as reversible dysfunction of RGCs and vision loss have been observed in animal glaucoma models [22, 73]. Second, purified RGCs models in vitro are well suited to investigate the intrinsic, cell autonomous aspects, but struggle to replicate the diverse interactions among different cell types in vivo, such as microglial cells, astrocytes and Müller cells [74, 75]. This necessitates the development of novel in vitro co-culture models or direct studies in animal models for a more accurate representation. Third, the recovery of dying/injured RGCs in our study is achieved by removing the cell death trigger. Glaucoma is a multifactorial disease, and while main treatments succeed in reducing IOP, degeneration often continues, leading to a further decline in patient vision [76, 77]. In situations where cell death stress cannot be removed, it becomes imperative to understand the mechanisms of RGCs recovery and to explore strategies, which improve the capacity of recovery, even in the presence of the cell death triggers.

## Conclusions

Our study on the (sub-)cellular level found that, by removing the cell death stimulus, injured RGCs could survive and recover from distinct mitochondrial damage and elevation of intracellular Ca^2+^, which occurred before the irreversible stage of massive cytochrome c release and caspase activation. These results may provide important information on how injured or dying RGCs can be rescued and may constitute the cellular basis of the clinically observed recovery of visual function after IOP reduction. Above all, a more in-depth analysis on the precise mechanisms that arrest the neuronal cell death pathway, repair the mitochondrial damage, and restore the normal mitochondrial network and function, would enhance our understanding of neurorescue, and provide new potential targets and therapeutic approaches for glaucoma. Furthermore, these neurorescue mechanisms may also have value for treatment of other neurodegenerative diseases, like Alzheimer’s disease and Parkinson.

## Material and methods

### Isolation of primary retinal ganglion cells and culture

All animal procedures were approved by the Central Authority for Scientific Procedures on Animals (CCD, Den Haag, NL), were approved by the local ethical committee, and were in accordance with the European Directive for animal experiments (2010/63/EU) (Approved Dutch license number: AVD10700202114405). They also complied with the Association for Research in Vision and Ophthalmology (ARVO) statement for the use of animals in ophthalmic and vision research. Female time-pregnant Sprague-Dawley rats were obtained from Central Animal Facility of Maastricht University. Primary rat RGCs (prRGCs) were purified from dissociated retinal tissue with an immunopanning-magnetic separation protocol, essentially as previously described [78], with minor modifications. The neural retina was taken out of the eyeballs of postnatal day 7 rat pups. Isolated retinas were incubated in Dulbecco’s Phosphate Buffered Saline (DPBS; SH30264.02, Cytiva) containing 20 units/ml of papain, 0.24 mg/ml of L-cysteine, and 10 units/ml of Dnase I to digest for 30 min at 37 °C. After digestion, the papain supernatant was aspirated, retaining the partially digested retinas. DPBS containing 1.5 mg/ml BSA and trypsin inhibitor was added to halt digestion, followed by a rabbit anti-rat macrophage antibody (CLAD51240, Sanbio). Then retinas were gently triturated to produce a single cell suspension. After allowing macrophage binding for 10 min, cells were pelleted by centrifuge and re-suspended in DPBS containing 6 mg/ml BSA and trypsin inhibitor. Cells were pelleted again and re-suspended in DPBS containing 0.2 mg/ml BSA and 0.1 mg/ml insulin. The cell suspension was filtered through an autoclaved 20 μm nylon mesh and then transferred to a 15 cm Petri dish that had been coated overnight with goat anti-rabbit IgG (H + L) (111-005-003, Jackson Immuno) in Tris-HCl (PH 9.5) and incubated for 30 min at room temperature to allow macrophages to adhere to the plate. Non-adherent cells were transferred to a second, identical 15 cm dish and incubated for 20 min to further deplete macrophages. Non-adherent cells were then gently shaken loose, collected, pelleted by centrifugation and re-suspended in RGC culture medium. Cells were then incubated with CD90.1 MicroBeads (130-121-273, Miltenyi Biotec) for 15 min at room temperature. After carefully washing, the cells were applied to a MS Column (Miltenyi Biotec) placed in a MiniMACS Separator (Miltenyi Biotec), then the retained cells were eluted as magnetic labeled RGC fraction. PrRGCs were cultured in modified DMEM-Sato base growth medium [78, 79] and incubated at 37 °C in humidified 5% CO_2_. Ibidi slides (80606) were used to culture prRGCs and pre-coated with PDL (50 μg/ml; P6407, sigma) and laminin (10 μg/ml; 3400-010-02, R&D Systems). Four pregnant rats and 30 pups in total were used in this study. For each RGC isolation, 1 pregnant rat was ordered. Depending on the amount of pups born, 6-10 pups were used for RGC isolation each time. From this pool of isolated RGCs, cells were distributed over experimental groups at random.

### HeLa cell culture

A HeLa cell line was purchased from ATCC. Cells were cultured in DMEM (Gibco™ 11960044) with 10% fetal bovine serum (FBS; F7524, Sigma-Aldrich), and 1% penicillin-streptomycin (Pen/Strep) antibiotic (Gibco™ 15140122), and incubated at 37 °C in a 5% CO_2_ incubator.

### Live cell imaging

For live cell imaging, we used FEI CorrSight microscope equipped with wide field and an Andromeda spinning disk. The FEI MAPS software, in conjunction with Live Acquisition software (LA, FEI) was used to control the microscope and to capture still and time-lapsed images. The microscope was further equipped with an incubation system able to control temperature at 37 °C (Ibidi) and CO_2_ at 5% (Digital Pixel, UK) at the specimen level. To monitor the cells over time, cell death stimuli were introduced to the cells through perfusion tubes (ibidi), which were connected to the cell chamber. To remove cell death stimuli, fresh medium was introduced to the chamber through these tubes. Externalization of phosphatidylserine along the plasma membrane of neurite was visualized by the fluorescent probe annexin A5-FITC (1 μg/ml, donated by Prof. Reutelingsperger’s lab; Biochemistry department, Maastricht University). Time-lapse imaging was used to record the progression of fluorescent signal along the neurite, corresponding to a wave of membrane lipid asymmetry along the neurite. Initial images were captured before ethanol treatment and then after adding ethanol, images were taken every 10 min with the laser line of 488 nm and a Semrock 446/523/600/677 nm BrightLine® quad-band bandpass filter until whole cells were stained with annexin A5-FITC. Z-stacks consisted of 10 planes with a Z-interval of 1 μm. Mock-treated cells were imaged in parallel to ensure that imaging and staining procedures were not cytotoxic. Images were showed in the merged z-stacks of 10 planes with the maximum fluorescence intensity. Representative images and videos were extracted and edited in Fiji software.

Mitochondrial structure and membrane potential were visualized with Mito-tracker (M22426, Invitrogen™) and TMRM (I34361, Invitrogen™) and imaged with laser line of 640 nm and 561 nm, respectively, and the 446/523/600/677 nm BrightLine® quad-band bandpass filter. Mitochondrial volume quantification was done with a similar MATLAB script as previously reported [80] with the modification that our analysis was performed in 3D using the DIPimage toolbox (https://diplib.org/). To quantify the mitochondrial volume, prRGCs were imaged with z-stacks consisting of 100 planes and an interval of 0.147 μm. Representative images were edited in Fiji or AmiraTM.

### Cell viability

Cell viability of prRGCs was determined by Hoechst 33342 (Sigma-Aldrich) and propidium iodide (PI, Sigma-Aldrich) double fluorescent staining as previously described [81]. Briefly, prRGCs were seeded on 6-channel μ-Slide (80,606, Ibidi) and cultured for 14 days. Cells were stained with Hoechst/PI and imaged with both fluorescence and bright field images under the FEI Corrsight microscope. Same cells were imaged after EtOH (5%, vol/vol, 3 h) treatment and after removing EtOH for another 20 h. At least 100 cells were imaged in each independent experiment. Quantification was done with Fiji.

### Correlative light and electron microscope (CLEM)

Cells were grown on a μ-Slide 8 Well Grid-500 (80826-G500, ibidi). For light microscope (LM) imaging, cells were observed under FEI Corrsight microscope equipped with Andromeda spinning disk. After LM imaging, cells were fixed with 2.5% Glutaraldehyde in 0.1 M phosphate buffer for 24 h at 4 °C. Then cells were washed with 0.1 M cacodylate buffer and postfixed with 1% osmium tetroxide in the same buffer containing 1.5% potassium ferricyanide for 1 h in the dark at 4 °C. Samples were dehydrated in ethanol, infiltrated with Epon resin for 2 days, embedded in the same resin and polymerized at 60 °C for 48 h. Ultrathin sections were cut using a Leica Ultracut UCT ultramicrotome (Leica Microsystems Vienna) and mounted on Formvar-coated copper grids, then stained with 2% uranyl acetate in water and lead citrate. Sections were observed in a Tecnai T12 Electron Microscope equipped with an Eagle 4kx4k CCD camera (Thermo Fisher Scientific, The Netherlands). Quantification of mitochondria related parameters from transmission electron microscopy (TEM) cross-sections, including mitochondrial length, perimeter, area, and cristae number per mitochondrion were done as previously reported [82] with Fiji.

### Immunofluorescence staining

Cells were fixed in 4% paraformaldehyde (PFA) for 30 min at 4 °C followed by permeabilization in 0.1% Triton X-100 for 10 min at room temperature and blocked with 4% Bovine Serum Albumin (BSA) for 1 h at room temperature. For immunostaining, cells were incubated with primary antibodies against cytochrome c (5 μg/mL; 33-8200, Invitrogen; RRID: AB_2533141) and TOM20 (1:50; 42406S, Cell Signaling Technology; RRID: AB_2687663) overnight at 4 °C. After carefully rinsing in PBS, cells were incubated for 1 h at room temperature with a second antibody conjugated with Alexa 488 and 594 (1:500, Invitrogen; RRID: AB_2633275, AB_2534079), respectively. The nucleus was labeled by DAPI (D9542, Sigma-Aldrich). The cells were then observed and photographed with the FEI CorrSight. Laser lines of 405, 488, and 561 nm in combination with the 446/523/600/677 nm BrightLine® quad-band bandpass emission filter were used to observe DAPI, cytochrome c, and TOM20, respectively.

### Intracellular Ca^2+^ measurement

The level of intracellular Ca^2+^ was measured using Fluo 8-AM (ab142773, Abcam) according to the manufacturer’s instruction. In brief, prRGCs were seeded on 6-channel μ-Slide (80,606, ibidi) and cultured for 14 days. Cells in control group and EtOH group (5%, 3 h) were incubated with 4 μM Fluo 8-AM diluted in medium for 30 min at 37 °C. Cells were then washed 3 times with culture medium and imaged under FEI CorrSight microscopy with the laser line of 488 and the 446/523/600/677 nm BrightLine® quad-band bandpass filter. Fluorescence intensity was quantified with Fiji.

### Statistical analysis

Data were obtained from at least three independent experiments and presented as mean ± SD or SEM. Statistical analyses were performed throughout using GraphPad Prism version 9.4.1. For the comparison between more than two groups, values were evaluated by one-way ANOVA followed by a Tukey Kramer test. *p* < 0.05 was considered statistically significant.

### Supplementary Information

**Additional file 1.**

**Additional file 2.**

**Additional file 3.**

**Additional file 4.**

## Data Availability

All data generated during this study are included in this article (and its supplementary information files).

## Abbreviations

RGC: Retinal ganglion cells
prRGCs: Primary rat retinal ganglion cells
PS: Phosphatidylserine
CNS: Central nervous system
IOP: Intraocular pressure
DARC: Detection of apoptosing retinal cells
EtOH: Ethanol
NC: Negative control
zVAD: Z-VAD-FMK
TMRM: Tetramethylrhodamine methyl ester
BDNF: Brain-derived neurotrophic factor
NGF: Nerve growth factor
CNTF: Ciliary neurotrophic factor
MOMP: Mitochondrial outer membrane permeabilization
STS: Staurosporine
CLEM: Correlative light and electron microscope
