# Supplementary material for: A time window for rescuing dying retinal ganglion cells
Source: Cell Commun Signal. 2024 Jan 31;22:88. doi: 10.1186/s12964-023-01427-3 (PMC10832163; doi:10.1186/s12964-023-01427-3)
Supplement: Supplementary file 2 — Additional file 1. [file 12964_2023_1427_MOESM1_ESM.docx]

**A Time Window for Rescuing Dying Retinal Ganglion Cells**

Wenting You ^1,2,3^, Kèvin Knoops ^4^, Iris Boesten ^1^, Tos T.J.M. Berendschot ^1^, Marc A.M.J. van Zandvoort ^5,6^, Birke J. Benedikter ^1^, Carroll A.B. Webers ^1^, Chris P.M. Reutelingsperger ^2,^* and Theo G.M.F. Gorgels ^1,^*

**Supplemental Figure 1**

**
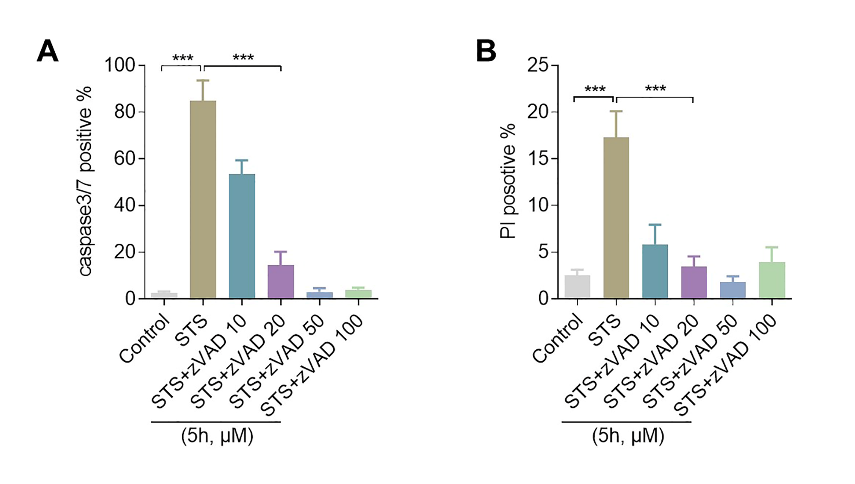
**

**Supplemental Figure 1. Pilot study of the working concentration of Z-VAD-FMK (zVAD).** The effect of caspase inhibitor zVAD on staurosporine (STS) induced caspase activation and cell death in HeLa cells. Cells were treated with or without STS (500 nM) for 5 h. To test the working concentration of zVAD, cells were pretreated with different concentration of zVAD (10, 20, 50, 100 µM) for 1 h, and then co-treated with STS (500 nM) for another 5 h. (**A**) Quantification of cells with caspase 3/7 activation measured by CellEvent™ Caspase-3/7 Detection probe (C10723, Invitrogen™) staining. (**B**) Quantification of cells positive with propidium iodide (PI) staining. Data are presented as the mean ± SEM. ****p* < 0.001.
